# Ten simple rules to cultivate belonging in collaborative data science research teams

**DOI:** 10.1371/journal.pcbi.1010567

**Published:** 2022-11-03

**Authors:** Kaitlyn M. Gaynor, Therese Azevedo, Clarissa Boyajian, Julien Brun, Amber E. Budden, Allie Cole, Samantha Csik, Joe DeCesaro, Halina Do-Linh, Joan Dudney, Carmen Galaz García, Scout Leonard, Nicholas J. Lyon, Althea Marks, Julia Parish, Alexandra A. Phillips, Courtney Scarborough, Joshua Smith, Marcus Thompson, Camila Vargas Poulsen, Caitlin R. Fong

**Affiliations:** 1 National Center for Ecological Analysis and Synthesis, University of California Santa Barbara, Santa Barbara, California, United States of America; 2 Departments of Zoology and Botany, University of British Columbia, Vancouver, British Columbia, Canada; 3 Bren School of Environmental Science and Management, University of California Santa Barbara, Santa Barbara, California, United States of America; 4 Main Library, University of California Santa Barbara, Santa Barbara, California, United States of America

This is a *PLOS Computational Biology* Methods paper.

## Introduction

*“If I get to be me, I belong. If I have to be like you*, *I fit in.”—Brené Brown, Braving the Wilderness.*

Data science is rapidly changing the landscape of scientific research. Research leveraging data science tools (hereafter, data science research) is increasingly widespread across disciplines, as software for large dataset analysis grows in power and reach [1–5]. With this expansion in data science research, coding and data analysis skills are becoming more valuable to early career researchers [6–8]. To ensure equitable participation in research and access to high-mobility career opportunities as this field expands, it is critical that data science research is welcoming and inclusive [9,10]. Beyond the moral imperative, improving inclusion will also advance the field, given that data science research is highly collaborative and team members bring diverse backgrounds and technical skills [5].

There are many cultural, economic, institutional, and social barriers to inclusion in academic research in general and data science in particular [11–13]. Science and data science have historically been dominated by members of identity groups with power and privilege, as marginalized identity groups were excluded from educational opportunities [12,14]. Systems of oppression have informed data science research practice in both subtle and overt ways, from a masculine workplace culture [15,16] to harmful terminology (e.g., “master/slave”; [17,18]). The world of data science research can also be exclusive and inaccessible due to the time, money, mentorship, or networks needed to learn data science skills [19]. These barriers are larger for members of historically excluded groups, who may additionally experience hiring and promotion discrimination, less access to quality education, and a lack of role models, encouragement, and self-confidence [20–22]. Dismantling these barriers will require large-scale structural change, both in research institutions and in society as a whole [10,23,24]. Here, we describe one way to reduce barriers while leveraging the strengths of diverse team members: **cultivate a sense of belonging in collaborative data science research teams**.

We define *belonging* as the feeling of deep connection to a community in which one is valued, accepted, and secure (adapted from [25–27]). Belonging is closely tied to psychological safety, or the feeling that one can express ideas, seek and provide feedback, and take risks without fear of rejection [28]. A sense of belonging among group members can promote collaborative, welcoming, and innovative research environments that foster the happiness, recruitment, and retention of diverse teams [29,30]. In turn, diverse research environments will further a sense of belonging, in a positive feedback loop with the added benefit of improving research outcomes [31,32]. With these 10 rules, our goal is to provide guidance to help activate this cycle across institutions and teams.

### Positionality and process statement

We are a team of researchers and data scientists across career stages based at the National Center for Ecological Analysis and Synthesis (NCEAS), an independent research affiliate of the University of California, Santa Barbara (UCSB). Our group includes Masters of Environmental Data Science (MEDS) students, members of the NCEAS executive team, postdoctoral researchers, science communicators, and staff scientists. To develop these rules, we drew on our collective experiences conducting team-based data-intensive science. Individually and collectively, we reflected on when we have and have not felt a sense of belonging, and what actions have, and have not, fostered that feeling. Throughout the manuscript, we cite many actions that we have found to be impactful at NCEAS, and while our examples draw heavily from our own experiences, the general rules apply across research environments.

Our perspectives and senses of belonging are shaped, in large part, by our individual experiences. We all have access to higher education and research opportunities and are based at UCSB, a space that is exclusive and privileged. While we represent a diversity of backgrounds and identities, including some that have been largely excluded from science, our authorship team of course does not reflect the full diversity of human experiences.

**Fig 1:**
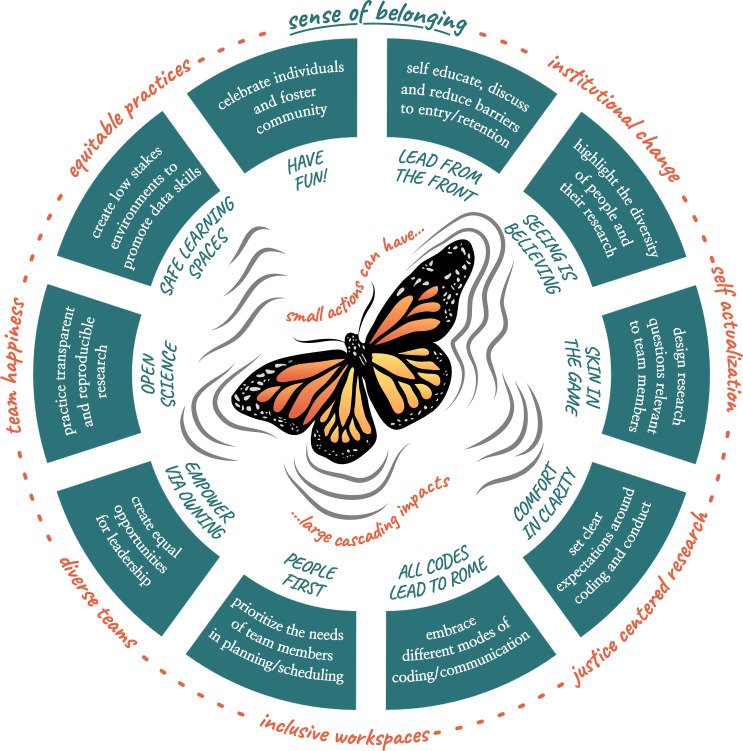
Ten simple rules to cultivate belonging in collaborative data science research teams, visualized here as the “butterfly effect”. Small actions taken by an individual or team, as outlined in our 10 rules, can generate a sense of belonging. This sense of belonging can then cascade to shape other aspects of culture and practice in science and beyond, as illustrated on the outside ring of words. *Illustration by A*.*A*.*P*.

Our NCEAS logo is a butterfly, inspired by Lorenz’s “butterfly effect”: the nonlinear equations describing how chaos is sensitive to initial conditions. As stated in Lorenz’s 1972 paper [33], “If the flap of a butterfly’s wings can be instrumental in generating a tornado, it can equally well be instrumental in preventing a tornado.” We use this theme as the inspiration for this paper: that small actions initiated by an individual can have cascading and amplifying effects across the world (Fig 1).

### Target audience

We hope that this guidance can be helpful for anyone leading a collaborative data science research project or initiative, including principal investigators (PIs), lead authors, project managers, and institutional leaders (henceforth, “leaders”). When we refer to “research teams,” we are referencing collaborative research teams that may be research labs, institutes, working groups, class project groups, or coauthors on a manuscript, within or outside of academia. While our recommendations are aimed at those in positions of power, we also believe that they can inspire advocacy for bottom-up change. We intend these rules to be a starting point and prompt all readers to reflect on actions they can take as individuals and teams to promote belonging within their research teams (and then take these actions!).

## Rule 1: *Lead from the front*: Self-educate, discuss, and reduce barriers to entry and retention in data science research

Open conversations about barriers to diversity and equity in data science can help to make team members feel seen and heard, especially when those in leadership positions initiate these conversations and then act upon them [9]. Leaders should educate themselves about how systemic and cultural barriers exclude people of marginalized identities from data science research [34]. As a leader, you can “lead from the front” by openly committing to diversity, equity, inclusion, and justice and normalizing team conversations about these topics to foster a sense of belonging [35]. At NCEAS, for example, we created a reading group that brought central leadership, research scientists, and staff together to collectively work through the publicly available Unlearning Racism in Geosciences (URGE) curriculum [36], which assigns readings and prompts for written deliverables in which we outlined anti-racist actions and policies for NCEAS, tailored to a data science context. NCEAS leadership then incorporated these deliverables into a public strategic plan that is updated every year with new perspectives from our evolving community. Similar action-oriented reading groups could guide meaningful, bottom-up change in other research teams. People in positions of power should not only increase awareness of barriers to entry and retention and their own positionality, but also use their own agency to reduce these barriers on their team. For example, it is common for student workers to be expected to purchase their own computing equipment, creating obstacles for students who do not have the resources to buy them. Inadequate funds or poor-performing computers create a sense of alienation and impede belonging. To mitigate these effects, leaders can allocate or apply for funds to purchase equipment, or provide equitable access to analytical servers with high computing power.

## Rule 2: *Seeing is believing*: Highlight the diversity of people, research, and accomplishments in data science

Individuals will experience a greater sense of belonging in data science research when they see themselves represented in the field [37]. To that end, teams should center and cite the work of people from diverse backgrounds in publications, presentations, training materials, and social media [38]. It is important to consider multiple axes of diversity in personal and professional identities, including career stage, disability, ethnicity, gender, institutional affiliation, immigration status, nationality, race, and sexual orientation (listed here alphabetically and nonexhaustively). That said, never reduce people to a single aspect of their identity, in or outside of your team (“tokenizing”; [39]), and steer clear of stereotypes and microaggressions [40]. At NCEAS, we host an annual seminar series in which a diverse group of speakers share their research approaches and findings as they relate to “Advancing Ecology and Environmental Data Science for a More Just and Equitable Future.” Given the lack of diversity in academia and data science, the visibility of role models from similar backgrounds can foster belonging [12]. You might, for example, encourage your team to join communities like Women in Data Science (WiDS) [41], Minorities in R [42], or PyLadies [43]. Within your research team, highlight and acknowledge the diversity of team members and the strengths they bring to the group and create a welcoming space for newcomers. For example, at NCEAS we have a #welcome Slack channel for newcomers to share a bit about their background with their colleagues and have established cultural norms around timely commenting and adding positive emojis [44]. Beyond Slack, we also welcome new hires by sharing a Google calendar page on which members of the community sign up for one-on-one coffee and lunches to build personal connections within and across teams. All team members can foster a sense of belonging by learning, recognizing, and respecting the identities of their members. For example, the display of names and pronouns can be accommodated in digital profiles and signatures.

## Rule 3: *Skin in the game*: Design research questions that are relevant to your research team members

Collaborative data science research benefits from focusing on questions that are aligned with the identity groups and communities to which team members belong. Consider how your data science team can adopt an intersectional lens [45], accounting for power structures among identity groups as you formulate research questions, compile and analyze data, and interpret findings. Where possible, the development of research questions should promote connections between data science and the lives, interests, and values of team members [46]. As a leader, you can make an active effort to consider how team members find meaning and purpose in their work, beyond the technical aspects of their roles. This relationship-building effort may involve conversations about an individual’s motivations, how their interests might contribute to the group’s overarching goals, and how day-to-day operations fit within larger actions and shared objectives [47]. You can also support individuals to explore questions of interest by stepping outside of their technical roles at times (e.g., inviting a data analyst to run a planning meeting, while providing guidance) and by giving them the opportunity to provide input on project decisions regardless of role or career stage [48]. Team members’ day-to-day operations, for example, may consist of meticulous coding, but linking these operations to larger collective goals that they helped to cocreate can cultivate belonging by positioning team members to have meaningful agency in their research.

## Rule 4: *Comfort through clarity*: Set clear expectations around coding practices and workplace conduct

With clearly defined expectations, team members can feel more secure in their roles and safe as members of a team. Your research team may create a manual and code of conduct for collaboration, coding, and ownership and credit practices, for example, as a README within a GitHub organization or an onboarding document for new members [49–51]. Clear guidance around research expectations and methods builds confidence in early career researchers who may be new to certain aspects of data science. This manual may also include a team culture and philosophy that centers inclusion [52]. Steps for reporting and conflict resolution should be accessible and transparent, shared with new hires, and be public on an institutional website, and codes of conduct should be used to hold people accountable. Transparent processes for handling reports and anonymous feedback can empower team members, should their sense of belonging or safety be threatened. At NCEAS, we have a code of conduct and reporting policy publicly posted on our website and share this document with all residents and visitors [53]. By engaging everybody in the creation of expectations and policies, you can foster a sense of belonging through participation and empower team members to hold each other accountable in a positive light, grounded in shared principles [23].

## Rule 5: *All codes lead to Rome*: Embrace different modes of coding and communicating

Team members will feel a greater sense of belonging when their individual work styles and contributions are recognized and valued. There are many different ways in which people best collaborate and communicate, and differences in individual and cultural backgrounds can shape these styles. By using multimodal communication methods that cater to this diversity (e.g., verbal, visual, written), your research team can best engage and support every individual, reduce obstacles like language barriers, and foster inclusion and belonging. For example, technology can be leveraged to create opportunities for participation across a range of modalities, including both verbal communication and written chats on conference software (e.g., Zoom), asynchronous comments on platforms (e.g., GitHub, Google Docs), messaging applications (e.g., Slack, Microsoft Teams, e-mail), or visual brainstorming tools (e.g., Mural, Google Jamboard). At NCEAS, we incorporate alternative mediums such as art to illustrate technical concepts (and beautify our physical spaces!; [54]). Teams should also celebrate a diversity of programming approaches and problem-solving strategies, recognizing that there is no single way to solve a particular computing challenge. Your team should also create avenues for horizontal communication among group members in different roles, and not just vertically between members and leaders [55]. For example, the Ocean Health Index team at NCEAS conducts weekly Seaside Chats where peer team members carve out time to meet separately from their PI to discuss coding challenges and share experiences [56].

## Rule 6: *People first*: Prioritize needs of team members in project scheduling and planning

The recognition and accommodation of team members as people with diverse physical, emotional, and mental needs can increase belonging [35]. When scheduling and planning data science research projects, it is best to have private conversations with team members about their needs and preferences [57]. For example, some people may prefer to manage their workload according to specific deliverables, while others prefer to know how much time they are expected to work. Some people work best with dedicated solitary time for deep work and focus, while others prefer varied tasks and regular team engagement. Leaders should speak to team members about their needs and set expectations accordingly [58]. When scheduling projects, recognize needs outside of the workplace, for example, by providing adequate leave for health and caregiving and ensuring that the culture allows for it to be taken [59]. At NCEAS, we do not assume that everybody works the same hours, and we schedule meetings through shared calendars where individuals are able to mark themselves as “busy” for personal reasons. Tools that support asynchronous work (e.g., Slack, GitHub) can also allow for greater scheduling flexibility on a daily basis. Teams should also ensure the accessibility of physical spaces, meeting platforms, and research materials to cater to all people (e.g., captioning of presentations, color-blind friendly palettes, gender-neutral bathrooms). At NCEAS, for example, we have a dedicated private room that can be used for personal or family-related needs, like therapy sessions or breastfeeding.

## Rule 7: *Empowerment through ownership*: Create opportunities for ownership, leadership, and development among all team members

By being empowered to take ownership of a particular task or initiative, team members will feel a greater sense of investment and belonging to the team. Leaders should take time to understand the unique professional goals and obstacles of each team member at their respective career stages [60,61]. This understanding is especially important given that the rewards systems for career growth will vary based on an individual’s professional goals (e.g., ownership of data and code may be important for data scientists, while authorship on peer-reviewed publications may be important for academics). Leaders should then structure work plans so that there are opportunities for individually tailored professional growth and development, for example, through delegated leadership of particular tasks or subprojects within the larger project. Team members can also rotate responsibilities and types of work tasks, for example, by taking turns leading meetings or designing code templates. In the MEDS capstone group projects at NCEAS, for example, every student had a designated leadership position while having the opportunity to rotate through tasks and roles related to client communication, meeting organization, and coding. Organizing coding-related tasks using GitHub Issues [62] and assigning or allowing team members to self-assign themselves to issues is another way to encourage and support ownership over project deliverables. These practices increase belonging on the team (and have added benefits for collaboration efficiency and skills development). Overall, a positive and inclusive team culture is one that creates opportunities for all members to succeed and celebrates their accomplishments.

## Rule 8: *Open science*: Practice transparent and reproducible research within and outside of your research group

Open science practices can improve communication and trust among a team and enable all team members to understand how they belong and how they can contribute [63,64]. “Open science” is conducted through transparent and accessible processes at all stages, including idea development, data collection, programming and analysis, and writing [65]. The use of GitHub “Issues” or “Discussions” features allows all members of a team to make coding decisions quickly and collaboratively [66]. In addition to well-documented project management, embedding ample comments, annotations, and explanations to the code and its documentation allows others to easily follow along. These practices also solidify institutional memory and can assist in onboarding new team members so that they are quickly brought up to speed on why decisions were made and can easily engage in future decision-making. It is worthwhile to invest time in training team members on these open science practices and software, both to improve open communication and to enable comfort with the tools and team. For example, the Openscapes initiative, based at NCEAS, provides structure to research teams implementing open science practices through the Openscapes Champions Program [67]. While open science has many merits for inclusion and for the scientific enterprise as a whole, one risk of open science is the failure to properly acknowledge the contributions of all who contribute to open scripts and datasets. To combat this risk and ensure a sense of belonging, your team should carefully consider citations and authorship to recognize contributions of all team members (aligning this recognition with individual career goals, as noted in Rule 7), as well as external contributors on whose work you build [68].

## Rule 9: *Safe learning spaces*: Create low-stakes environments to promote data science skills growth

Normalizing the vulnerability of not knowing or understanding something is vital for fostering psychological safety and an inclusive research environment [69]. To that end, judgment-free spaces should be created for asking questions about research and coding [56,70] and where team members can work toward developing and growing their abilities [71]. At NCEAS, we hold casual coworking “hacky hours” where individuals share a coding issue that they are working through and get support and feedback from the group as they tackle it. In group meetings, asking questions can be encouraged by, for example, giving others the chance to “+1” questions so that people realize they are not alone in needing clarity and reinforcing the benefit of question-asking for the entire team. Regular and informal peer-led skills training can also provide a low-stakes venue for learning [70]. For example, our NCEAS early career research staff leads local R-Ladies [72] and Python meetup events, workshops through the EcoDataScience group [73], and coding book clubs. By sharing in the learning process with peers and colleagues across career stages, scientists can feel a greater sense of belonging as they fill knowledge gaps [74]. Leaders should also give feedback on work in a kind and constructive manner and emphasize mutual learning regardless of seniority [75]. Also, openly demonstrating struggles and failures (like talking through programming errors while live coding, or discussing unsuccessful submissions of publications and grants) along with sharing successes normalizes vulnerability [76]. By sharing small and large setbacks in informal and formal settings (e.g., presentations, group meetings), those in more senior positions can help to reduce doubts and insecurities among career team members [77].

## Rule 10: *Have fun*!

The implementation of the rules above requires thought and consideration, but that doesn’t mean your team cannot have fun at the same time. Many of the actions above can be really enjoyable for everybody involved, and creating fun spaces where people can bring their full selves can further contribute to a sense of community and belonging. Data science can be challenging and frustrating, especially for people just learning to code and people who feel marginalized, and having fun is critical to overcoming frustration [78]. Further, the seriousness and rigor of data science, often a characteristic of academia, can involve implicit norms about who gets to be a researcher and create barriers to belonging [79]. Having fun can build community and trust and provide a supportive environment for breaking down coding blocks. Through both targeted “fun” initiatives and informal workplace practices, your data science team can foster silly traditions, make use of humor, “gamify” coding, and create space for personal interaction. Make space for the diversity of ways that people have fun, and reduce barriers to participation in the fun—for example, provide context for idioms and pop culture references [80]. At NCEAS, we have regular social hours with and without alcohol, collect “hex stickers” as *R* packages are learned [81], engage in coding challenges, and make ample use of emojis and memes on our Slack channels to build skills and relationships while having fun. Overall, fun has been linked to benefits for learning, health, innovation, and creativity—so, have fun!

## Conclusions

Creating more inclusive data science research spaces and experiences is an ongoing process that is best cocreated and regularly reassessed by teams. We present these 10 “simple” rules with the understanding that these recommendations may not resonate with everybody and that there are far more than 10 ways to foster a sense of belonging. These recommendations are neither simple nor exhaustive. This is not a checklist on the path to inclusivity. We intend for these rules, based on our own experiences, to spark reflection and conversation rather than provide a universal roadmap to belonging. We look forward to continued conversations about ways to advance inclusion and belonging in collaborative data science research and beyond. To that end, we are excited to hear others share their advice and experience on Twitter, using the hashtag #BelongingInDataSci.
